# Cost of health inequality to the NHS in Wales

**DOI:** 10.3389/fpubh.2022.959283

**Published:** 2022-09-16

**Authors:** Rajendra Kadel, James Allen, Oliver Darlington, Rebecca Masters, Brendan Collins, Joanna M. Charles, Miqdad Asaria, Mariana Dyakova, Mark Bellis, Richard Cookson

**Affiliations:** ^1^WHO CC on Investment for Health and Wellbeing, Public Health Wales, Cardiff, United Kingdom; ^2^Health and Social Services Group, Finance Directorate, Welsh Government, Cardiff, United Kingdom; ^3^Department of Health Policy, London School of Economics and Political Science, London, United Kingdom; ^4^Centre for Health Economics, University of York, York, United Kingdom

**Keywords:** cost, inequality, health service use, NHS, Wales

## Abstract

**Background:**

Forty years from the seminal work of Welsh GP Julian Tudor Hart on the Inverse Care Law, inequalities in health and healthcare remain deeply embedded in Wales. There is a wider gap (over 17 years) in healthy life expectancy between people living in the most and least deprived neighborhoods in Wales. This health inequality is reflected in additional healthcare use. In this study we estimate the cost of inequality associated with this additional healthcare use to the publicly funded National Health Service (NHS) in Wales.

**Methods:**

We retrieved administrative data on all NHS inpatient admissions, outpatient and accident and emergency attendances in Wales between April 2018 and March 2019 from Digital Health and Care Wales (DHCW). Hospital service use data were translated to costs using Healthcare Resource Group (HRG) and health service specific unit cost data and linked with area level mid-year population and deprivation indices in order to calculate the healthcare costs associated with socioeconomics deprivation.

**Results:**

Inequality in healthcare use between people from more and less deprived neighborhoods was associated with an additional cost of £322 million per year to the NHS in Wales, accounting for 8.7% of total NHS hospital expenditure in the country. Emergency inpatient admissions made up by far the largest component of this additional cost contributing £247.4 million, 77% of the total. There are also substantial costs of inequality for A&E attendances and outpatient visits, though not maternity services. Elective admissions overall have a negative cost of inequality, since among men aged 50–75 and women aged 60–70, elective utilization is actually negatively associated with deprivation.

**Conclusion:**

There are wide inequalities in health and healthcare use between people living in more deprived neighborhoods and those living in less deprived neighborhoods in Wales. Tackling health inequality through a combination of health promotion and early intervention policies targeted toward deprived communities could yield substantial improvement in health and wellbeing, as well as savings for the Welsh NHS through reduced use of emergency hospital care.

## Introduction

Population health and wellbeing are substantially influenced by wider socio-economic (such as age, sex, income, education level etc.) and environmental factors (such as poor housing, poor working conditions etc.) (1). There are large inequalities in health and health service utilization between more and less socially advantaged groups (2). This results in different levels of healthcare needs which require relevant access to and provision of healthcare services. Health inequality has been a longstanding issue in Wales: people living in the most deprived neighborhoods (as measured by quintile groups) have a much higher probability of death from avoidable causes (3.7 times for males and 3.8 times for females) (3), and much worse health with fewer years of healthy life expectancy at birth (16.9 years for males and 18.3 years for females) compared to those living in the least deprived neighborhoods (4). People living in more deprived neighborhoods tend to consume more emergency healthcare resources at any given age, in terms of volume and cost, as they experience more adverse health conditions (5). However, although people living in more deprived neighborhoods are more likely to use emergency care [non-elective admissions, emergency GP appointments and accident and emergency (A&E) attendances] they are less likely to use specialist and preventative services, while the social patterning of elective inpatient care and primary care varies (6).

Disparities in healthcare service use can have a huge economic impact both in the short and the long-term. In EU, the annual cost of health inequality was estimated to be €980 billion or 9.4% of GDP (7). In UK, the cost of inequality in health service use was estimated to be £4.8 billion per year to the NHS in England (8) using data from 2011. The economic cost related to inequality in healthcare use has not been established for Wales specifically, or updated to recent years. This study aims to estimate the social gradient in different categories of hospital utilization and estimate the overall cost to the NHS in Wales of socioeconomic inequalities in health.

## Methods

We used whole-population hospital service data to estimate hospital utilization and cost by five deprivation groups for different age groups, sex and hospital service types. The cost of health inequality to the NHS in Wales was defined as the excess hospital utilization costs among the most deprived four-fifths of the population compared with the least deprived fifth of the population.

### Population and data source

The resource use analysis was restricted to Welsh residents who visited either NHS hospitals or NHS funded private hospitals (including those who received hospital services in England) for inpatient admissions, outpatient appointments and accident and emergency attendances. The hospital service datasets included information about the patient's age, sex, area of residence, and their status of health service use, including diagnosis, procedures and frequencies.

Three different hospital service use datasets—hospital inpatient episodes, outpatient appointments and accident and emergency (A&E) attendances—were retrieved from the Patient Episodes Dataset for Wales (PEDW) managed by Digital Health and Care Wales (DHCW) for 1 year period between April 2018 and March 2019. In general, the data are regularly collected and coded at each hospital and then these records are electronically transferred to the DHCW, where data are validated and merged into the main database. Finished Consultant Episodes (FCE) were used to estimate the number of hospital inpatient episodes which are allocated to a Healthcare Resource Group (HRG) code. Healthcare Resource Groups (HRGs) are clinical groupings of treatment activities according to procedures and diagnoses, which consume similar healthcare resources (9). Cost information for the financial year 2018/19 was collected from the Finance Delivery Unit (FDU) hosted by Public Health Wales. The cost information consisted of HRG reference costs for inpatient episodes and unit cost per visit for outpatient appointments and A&E attendances. The population data required for the analysis at population level was drawn from the mid-year population estimates, supplied by the Office for National Statistics (10).

The researchers collected anonymized patient level data and do not have access to patient identifiable codes. The researchers fully comply with GDPR 2018 to handle such data.

### Measurement of outcomes and costs

We estimated the total cost of health inequality according to hospital service types, sex and different age groups. Moreover, we also included social patterns of hospital episodes and attendances, and average cost according to age, sex and levels of deprivation (quintiles) for different hospital service types. We classified patients' age to represent meaningful age groups: infants (<1 year old), under 5 children, under 16 children, working age group (16–64 year olds), older age group (65 year olds and above), and maternity age group (15–44 year olds).

The basic geographical unit of analysis in this study is the Lower-layer Super Output Area (LSOA). Wales is divided into 1,909 LSOAs, each consisted of around 1,500 individuals on average. Area-based deprivation for LSOAs are measured using the Welsh Index of Multiple Deprivation (WIMD) for 2019. WIMD comprised of seven domains: income, employment, health and disability, educational skills and training, barriers to housing and services, crime, and living environment. These domains are combined to produce an overall deprivation rank for each LSOA. In this study, we used LSOA grouping into quintiles based on deprivation ranks, ranging from Q1 (the least deprived fifth of LSOAs) to Q5 (the most deprived fifth of LSOAs) (11).

Hospital service categories were divided into inpatient admissions, outpatient and accident and emergency attendances. Hospital inpatient admission was further divided into elective, emergency and maternity admissions (12). In elective inpatient admission, a patient whose admission date is known in advance thus allowing arrangements to be made beforehand. However, patient's admission date and time is unpredictable and at a short notice due to its clinical need in case of emergency inpatient admission. If a hospital admission of a pregnant or recently pregnant woman to a maternity ward is done, except when the intention is to terminate pregnancy, this is called maternity inpatient admission. If an appointment is made at a hospital outpatient department or clinic for the purpose of consultation, examination or treatment by a doctor or independent nurse, this is called an outpatient appointment. Similarly, an accident and emergency (A&E) attendance is the one when a patient visits an accident and emergency department to receive advice and treatment for patients with urgent healthcare need.

The outcome measures in this study were the episodes and frequency of hospital service use (inpatient admissions, outpatient and A&E attendances) recorded in hospital administrative database. Cost of inpatient stays, outpatient, and A&E attendances were estimated using hospital episodes statistics and the Welsh reference cost data received from the FDU. The cost for the health service use was reported in 2018 pound sterling (£) from the NHS Wales perspective.

### Statistical analysis

Hospital service use data were grouped into age, sex and WIMD quintile categories. Then total costs for each age, sex and WIMD quintile group were computed using the HRGs and the relevant reference costs application to hospital inpatient, outpatient and A&E cases. This analysis used a simplified version of health service use and cost estimation, which was scaled up using patient population numbers and disaggregated the population-level results according to the social patterns observed in the health service use data. In brief, we estimated the average cost (per person) in each age, sex and levels of deprivation according to WIMD quintile by dividing the total costs by the mid-year population using ONS population estimates.


$$Average\;Costs_{Age,\;Sex,\;WIMD^{\;}}=\frac{\sum Hospital\;Costs_{Age,\;Sex,\;WIMD}}{\sum opulation_{Age,\;Sex,\;WIMD}}$$

The annual cost of health inequality was computed by multiplying the difference between total average annual cost and average annual cost of the least deprived group by mid-year population. The cost of health inequalities were also computed separately for specific age groups, sex and hospital service types.


$$\begin{array}{l} Cost\;of\;Inequality_{Age,\;Sex,\;WIMD}\\ \;=\sum [Population_{Age,\;Sex,\;WIMD}\\ \;\times (Average\;Costs_{Age,\;Sex,WIMD\;=\;Q1-5}\\ \;-Average\;Costs_{Age,\;Sex,\;WIMD\;=\;Q1})] \end{array}$$

We used Microsoft SQL Server Management Studio 17 and Excel 2016 for data analysis.

## Results

Results are presented into three categories: social patterns of hospital service use, cost of health inequality, and average cost of hospital service use.

### Social patterns of hospital service use

Table 1 presents the social patterns of hospital service use according to sex, levels of deprivation and hospital service types. A total of 1.22 million hospital impatient episodes, 4.4 million outpatient appointments, and 0.87 million accident and emergency attendances were made between April 2018 and March 2019. The median age of patients receiving hospital service was the highest for elective admissions (63 years) followed by emergency admissions (62 years).

**Table 1:** Social pattern of hospital service use by age, sex and level of deprivation, Wales 2018/19.

	**Hospital inpatient episodes**			**Outpatient**	**A&E**	**Population**
**Characteristics**	**Elective**	**Emergency**	**Maternity**			**(Mid-year 2018)**
N	564,550	572,667	83,573	4,453,343	870,362	3,138,631
Age (median)	63 years	62 years	29 years	56 years	39 years	42.5 years
**Sex**						
Male	276,584 (49.0%)	276,043 (48.2%)	N/A	1,941,852 (43.6%)	436,591 (50.2%)	1,547,309 (49.3%)
Female	287,945 (51.0%)	296,607 (51.8%)	83,573 (100.0%)	2,511,491 (56.4%)	433,771 (49.8%)	1,591,322 (50.7%)
**Welsh index of multiple deprivation quintile (2019)**						
Q1 (Least deprived)	114,544 (20.3%)	90,525 (15.8%)	13,950 (16.7%)	860,237 (19.3%)	119,491 (13.7%)	623,358 (19.9%)
Q2	118,585 (21.0%)	105,046 (18.3%)	13,742 (16.5%)	894,317 (20.1%)	164,208 (18.9%)	638,695 (20.4%)
Q3	114,761 (20.3%)	112,173 (19.6%)	14,642 (17.5%)	866,161 (19.5%)	180,290 (20.7%)	643,563 (20.5%)
Q4	110,618 (19.6%)	125,844 (22.0%)	17,403 (20.8%)	910,571 (20.5%)	202,670 (23.3%)	618,030 (19.7%)
Q5 (Most deprived)	106,021 (18.8%)	139,062 (24.3%)	23,778 (28.5%)	922,057 (20.7%)	203,703 (23.4%)	614,985 (19.6%)

N/A, not applicable.

There was no substantial difference in percentage of hospital service use between males and females across different hospital service categories. In terms of levels of deprivation, there were wider differences in service utilization between people living in the most deprived neighborhoods and those living in the least deprived neighborhoods for A&E attendances (23.4 vs. 13.7%), emergency admissions (24.3 vs. 15.8%), and maternity admissions (28.5 vs. 16.7%).

### Cost of health inequality

#### Cost of health inequality by hospital service categories and sex

**Figure 1:**
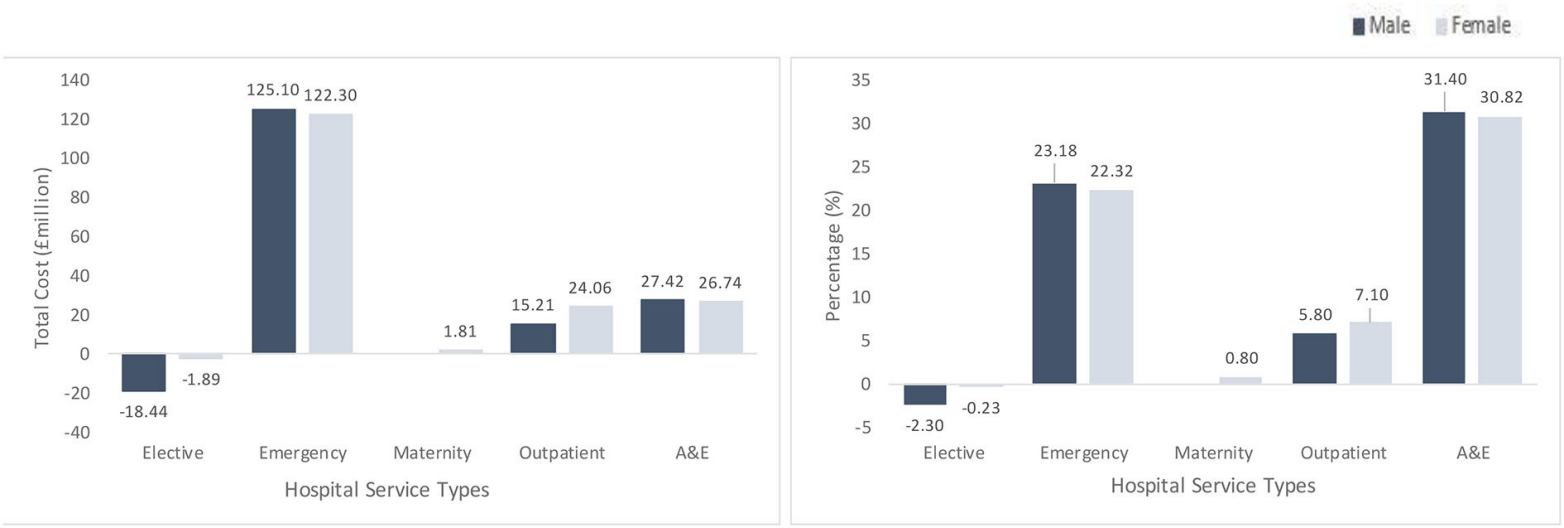
Total (left) and proportional (right) cost of health inequality. Left graph shows the total cost of health inequality in million (£) and right graph shows the proportional (%) cost of health inequality. x-axis in both graphs show hospital service types [elective inpatient, emergency inpatient, maternity inpatient, outpatient appointment and accident and emergency (A&E) attendance] disaggregated by sex with males in dark blue and females in light blue color.

The total cost of health inequality in hospital service use is estimated to be £322 million in Wales, which is equivalent to 8.7% of the total hospital expenses. Emergency admissions are the largest contributor to the overall cost of health inequality, with an additional cost of £247.4 million annually (Figure 1). Within service categories, A&E bear the highest proportional cost of health inequality (31%), followed by emergency admissions (23%).

Elective inpatient admissions have a negative overall and proportional cost of inequality, indicating that people from the least deprived neighborhoods have higher levels of elective inpatient utilization compared to those in more deprived neighborhoods.

#### Average cost (per capita) of health inequalityby hospital service categories, sex,and age group

**Figure 2:**
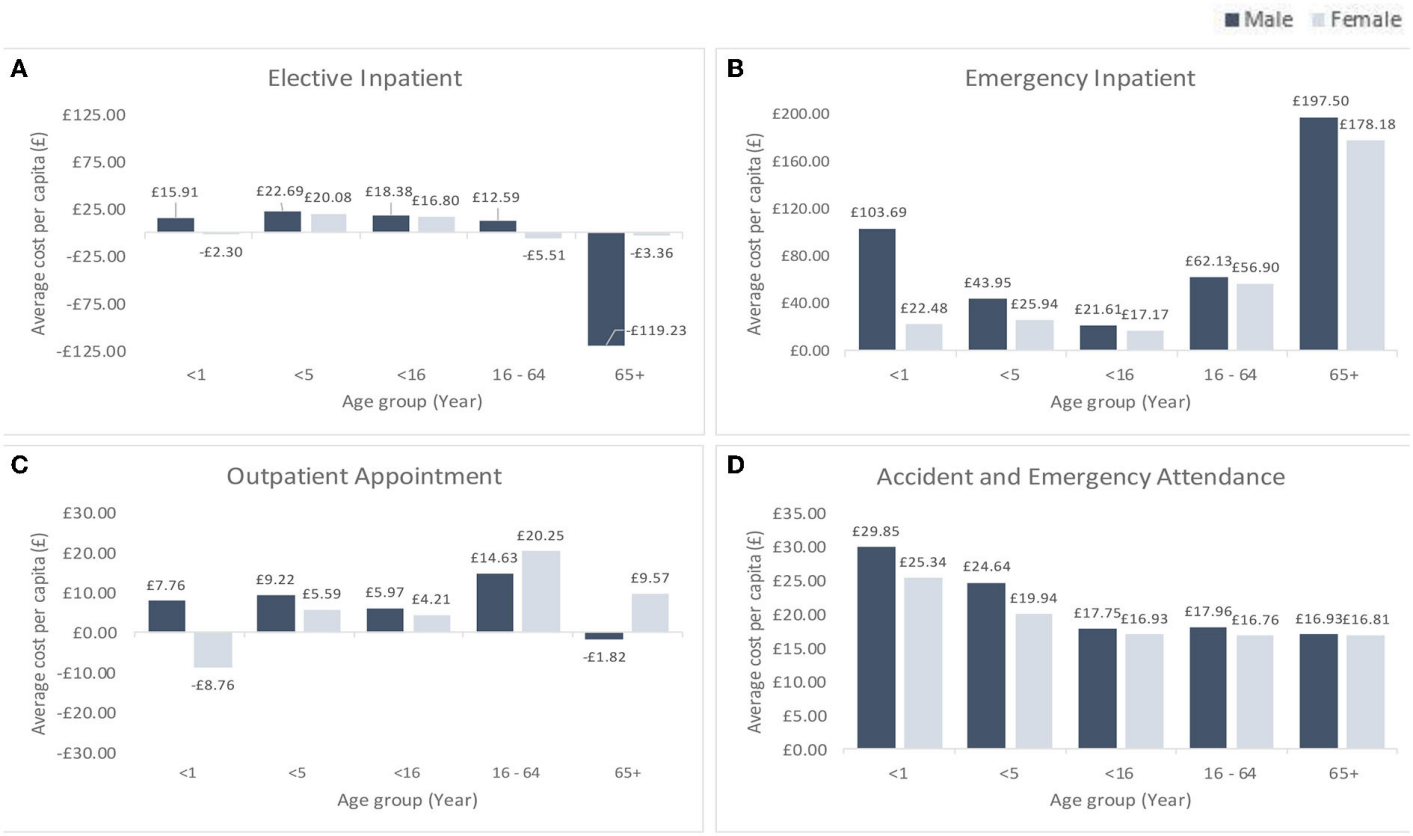
Cost of health inequality per capita by hospital service types, sex and age group. This figure is based on hospital episodes and attendance statistics for 2018/19 financial year in Wales. Results in the graphs are broken down by age groups—Infant (<1 year olds), under 5 year olds, under 16 year olds, 16–64 year olds (working age), 65 year olds and above (old age), by sex (Dark blue color bar charts are for males and light blue for females), and by hospital service types (**A**, Elective Inpatient; **B**, Emergency Inpatient; **C**, Outpatient Appointment; and **D**, Accident and Emergency Attendance).

For elective admissions, males aged 65 years and above incurred the highest negative cost per capita of health inequality (–£119 per person), resulting in an overall negative cost of health inequality attributed to the socioeconomic deprivation in this service category (Figure 2). There was also a substantial disparity in cost per capita of health inequality between males and females in this age category (–£119 vs. –£3.36). For emergency inpatient, the highest average cost per capita of health inequality were incurred by older age group (£197.50 for males, and £178.18 for females) and the lowest by under 16 age group (male: £21.61 and female: £17.17).

Average costs per capita of health inequality for outpatient appointments were the highest among working age people (male: £14.63 and female: 20.25). There were substantial differences in cost per capita of health inequality among infants (male: £7.76 and female: –£8.76) and older age group (male: –£1.82 and female: £9.57) in this service category. For A&E attendances, the average cost per capita of health inequality was the highest for infants (male: £29.85 and female: £25.34) and the lowest for older age group (male: £16. 93 and female: £16. 81).

#### Average costs of hospital service use

##### Elective inpatient admissions (upper charts)

**Figure 3:**
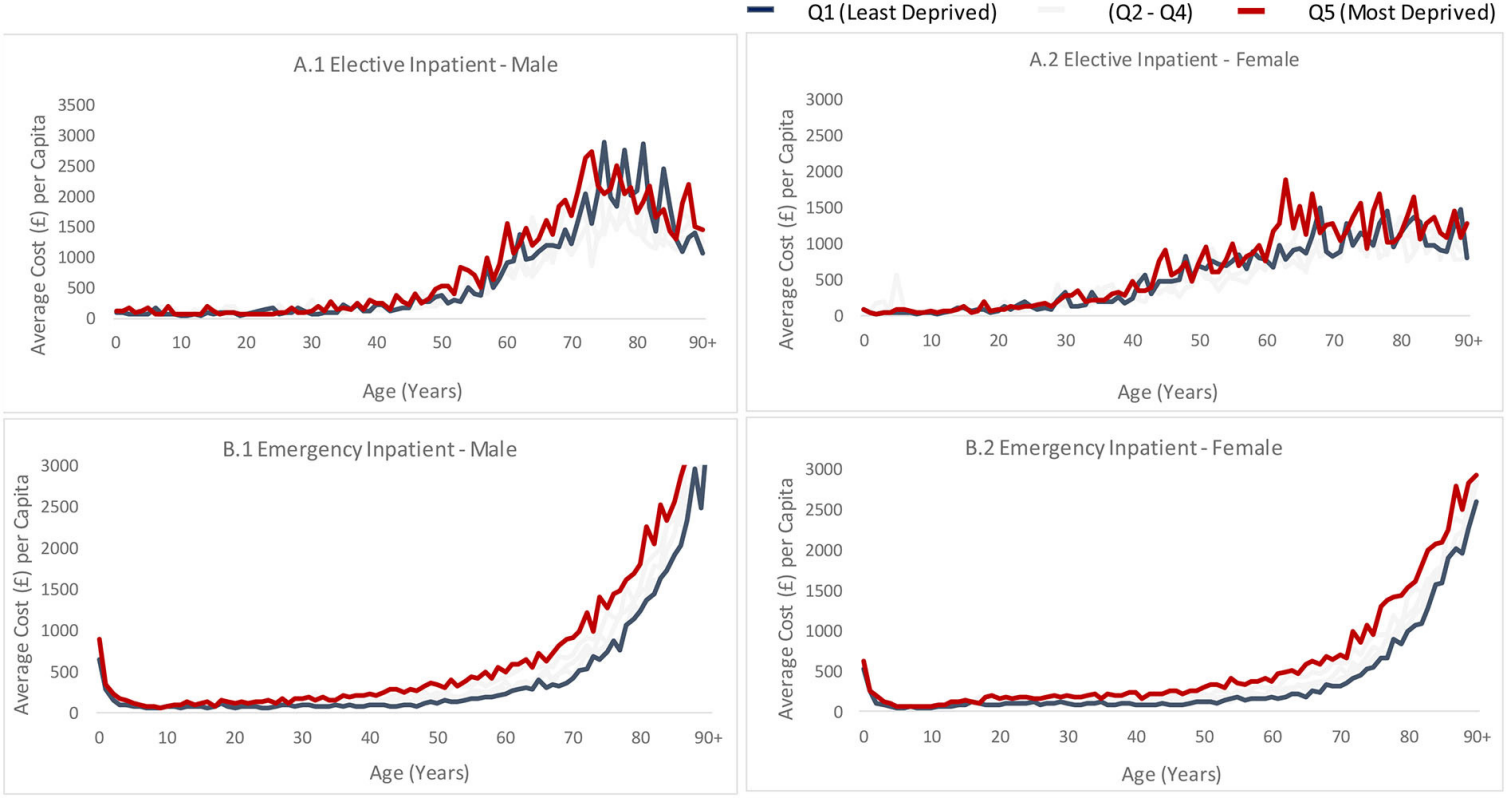
Average cost of inpatient service by age, sex and deprivation quintile. Graphs are based on Welsh' hospital episode statistics for 2018/19 financial year and are split by sex (male on the left and female on the right), deprivation quintile (different line colors) and are plotted against age (year). **(A.1)** Shows average elective inpatient cost for males, **(A.2)** shows average elective inpatient cost for females, **(B.1)** shows average emergency inpatient cost for males and **(B.2)** shows average emergency cost for females.

The elective inpatient cost per person is higher for both males and females living in the most deprived neighborhoods (16.0 and 18.0% higher, respectively) as compared to those in the least deprived neighborhoods (Figure 3). There is no clear social gradient across different age groups for both sexes.

##### Emergency inpatient admissions (Figure 3 - lower charts)

The annual emergency inpatient cost per person is much higher for both males and females living in the most deprived neighborhoods (57.0 and 62.0% higher, respectively) compared to those living in the least deprived neighborhoods (including costs for all ages). There is a clear social gradient for those at the age of 40 and above for both sexes.

##### Outpatient Appointments (upper charts)

**Figure 4:**
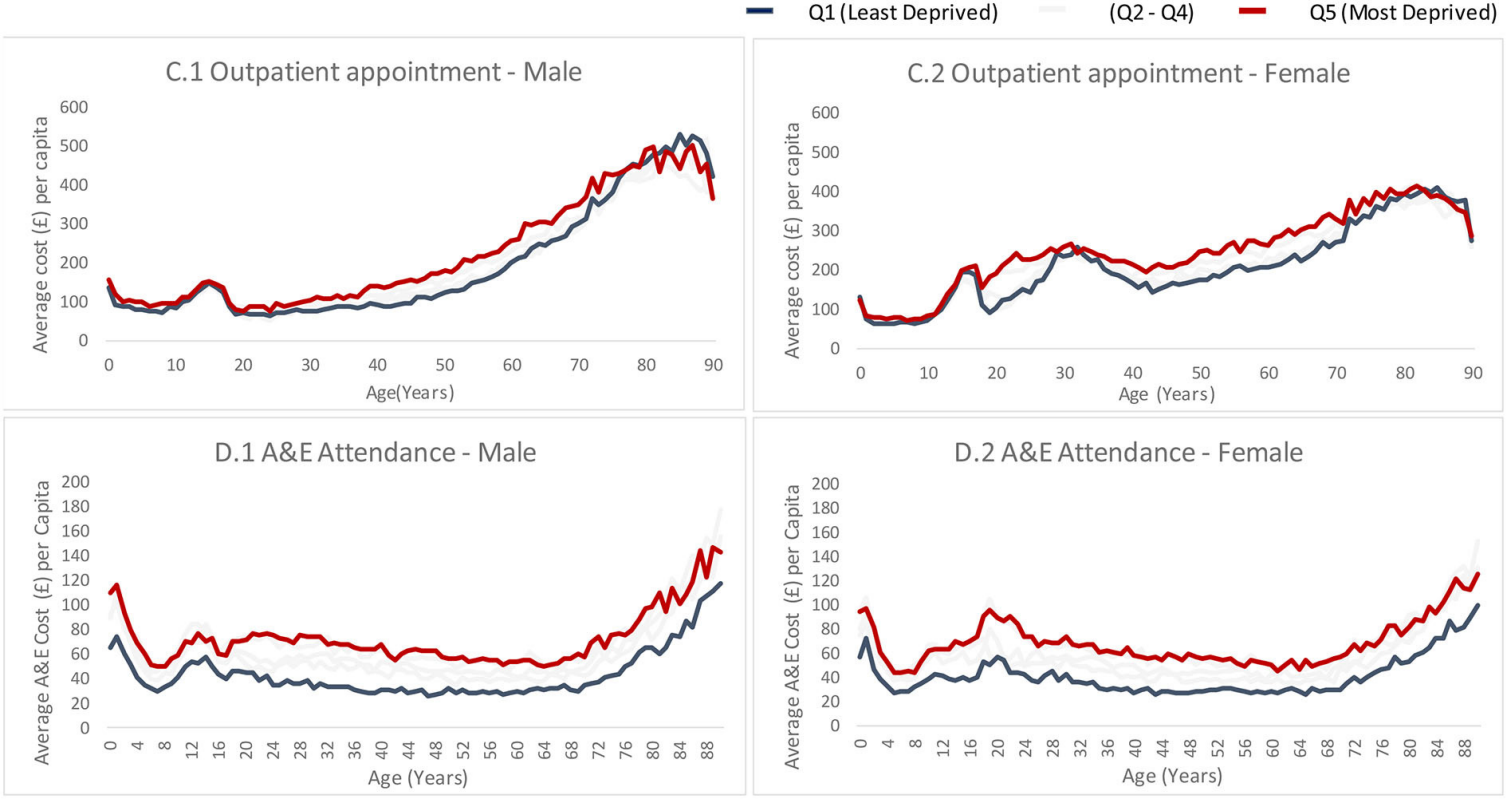
Average cost for outpatient and A&E attendances by age, sex and deprivation quintile. Graphs are based on Welsh' hospital attendance statistics for 2018/19 financial year and are broken down by sex (male on the left and female on the right) deprivation quintile (different line colors) and are plotted against age. **(C.1)** Shows average outpatient appointment cost for males, **(C.2)** shows average outpatient cost for females, **(D.1)** shows average accident and emergency (A&E) attendance cost for males and **(D.2)** shows average A&E attendance cost for females.

The average outpatient cost is slightly higher for both males and females living in the most deprived neighborhoods (14.0 and 18.0% higher, respectively) compared to those living in the least deprived neighborhoods Figure 4. There is a clear social gradient in the middle age groups for both sexes. There is a wider difference in cost between the most and the least deprived among younger females (18–28 years) compared to same age males.

##### A&E attendances (lower charts)

The annual A&E cost per person is much higher for both males and females living in the most deprived neighborhoods (67.0 and 68.0%, respectively) compared to those living in the least deprived neighborhoods (including costs for all ages) Figure 3. There is a more prominent social gradient in the middle age groups for males, compared to same age females. There is a wider difference in cost between the most and the least deprived among younger females (18–28 years) compared to same age males.

## Discussion

### Summary of findings

The aim of the study was to estimate the cost of health inequality associated with socioeconomic deprivation in hospital service use to the NHS in Wales. The study findings showed that the total cost of health inequality to the NHS in Wales is estimated to be £322 million, equivalent to 8.7% of the total hospital service expenses, in 2018/19. Emergency inpatient admissions are the largest contributor to the overall cost of health inequality, with an additional cost of £247.4 million annually. Average cost per capita of health inequalities varies widely among different age groups, sexes and health service types. For emergency inpatient, the average cost per capita of health inequality among older people was substantial, but there was substantial average negative cost per capita of health inequality for elective inpatient among older males. We also found that there is wider gap in cost of health inequality between sexes among older age group, indicating older people from less deprived areas has been receiving more health services. One of the possible reason behind such gap may be due to poor health seeking behavior among older males living in more deprived areas compared to people living in less deprived areas for elective admissions. We also noted a wider gap in cost of health inequality among children under 1 year. This may partly be due to more health problems in male infants living in more deprived areas.

Average cost per capita of health inequality was higher among working age group for outpatient appointments, while the trends in average cost per capita of health inequality was decreased with age groups for A&E attendances. We also found that the levels of healthcare costs increased with age, and were higher among females in their reproductive years, and higher in males at most other ages. The average healthcare cost aggregated across all service categories is higher for both males and females living in the most deprived neighborhoods as compared to those in the least deprived in every age group. There is also a clear social gradient in average costs of health service use within each service category with the exception of elective admissions.

The concept of “Inverse Care Law” theorized by visionary Welsh GP Julian Tudor Hart in 1971 stated that “The availability of good medical care tends to vary inversely with the need for it in the population served.” This study does not directly measure the inverse care law—it does not look at the quality of care, and it does not look at primary care (which is what Tudor Hart was mainly concerned about) (13). Rather, this study looks at the consequences of the inverse care law for the costs of secondary care (including emergency care), using hospital data not available in Tudor Hart's time.

In England and other high income countries with universal healthcare systems, we only have a disproportional care law these days where people from socially disadvantaged communities receive more healthcare, but provision of such care are poor in quality and are inadequate to meet their healthcare needs (8, 14). Disproportional care law is also evident in Wales for emergency inpatients, outpatient appointments and A&E attendances, but that this is shocking evidence of an actual inverse care law emerging for elective hospital care whereby poorer people receive less care despite being less healthy and having greater needs for care. As a consequence of this, for these poor people are not accessing inpatient care to the same extent as similarly sick rich people, their health problems are therefore not tackled early on when they are relatively cheap and easy to deal with to the same extent as for similarly sick rich people. Instead these health problems in poor people are more likely to develop into more serious conditions that require more extensive treatment when they come in as more expensive emergency admissions.

We define the cost of health inequality in terms of disparities in hospital service utilization across deprivation quintiles, which in turn are driven by wider socioeconomic inequalities. The fact that health service utilization differs across social groups has been demonstrated consistently for over 70 years. Writing in 1968, the founding chair of the London School of Economics noted that “We have learnt from the 15 years' experience of the health service that the higher income groups know how to make better use of service; they tend to receive more specialist attention; occupy more of the beds in better equipped and staffed hospitals; receive more elective surgery; have better maternal care, ad are more likely to get psychiatric help and psychotherapy than low-income groups particularly the unskilled (15).”

### Strengths and limitations

This is the first study from Wales using whole population data to estimate the cost of health inequality associated with socioeconomic deprivation to the NHS in Wales. However, there are several limitations that should be considered. First, we only look at associations between healthcare costs and deprivation, and do not perform causal inference analysis. It is reasonable to assume that deprivation causes excess healthcare costs rather than the other way around, however, since the NHS provides universal care that is free at the point of delivery and the incidence of catastrophic health care costs in the UK is extremely low by international standards. Second, we only look at the costs of health inequality not at the costs and effectiveness of interventions to tackle health inequality. Interventions to reduce health inequality would yield savings to the NHS from reduced emergency admissions, but they might also impose costs of various kinds and we cannot draw conclusions about the likely overall balance of costs and savings. Third, the population data used in this study were truncated at 90 years of age, assuming that the health service use remains constant at older age groups (90+). Fourth, this study uses the Welsh Index of Multiple Deprivation (WIMD) to categorize population into five quintile groups of small areas. The index is an area-based measure, which means that in this analysis, inferences about the nature of individuals are deduced from inferences about the area, e.g., an individual may live in a neighborhood that has a high level of deprivation, but that does not necessarily mean the individual has low income or education or other individual-level markers of social disadvantage. One of the domains of the WIMD is health but none of the data sources for this domain are around hospital admissions, however there may still be a small element of circularity with this analysis. Fifth, this study focuses on the cost of inequality in hospital service utilization, estimated according to service category and by age, sex and level of deprivation. Other services, such as primary and social care, and community health services might have an impact on the uses and economic costs of health services, as well as incur additional cost of health inequality. These might lead to under-estimation of the total cost of health inequality.

### Existing evidence to support this study

The current study results support and expand findings from the previous studies. Asaria, Doran (8) conducted a study based on comprehensive whole population data to explore the relationship between hospital costs and socioeconomic inequality in England. Their study found that inpatient hospital costs were 31% higher for people living in the most deprived neighborhoods compared with those living in the least deprived. We also used the similar approach, but our study was based on latest hospital service use data from Wales and estimated the cost of health inequality by including a broader group of hospital services, including outpatient and A&E attendances as well as inpatient admissions, and splitting out maternity admissions. Our study found wider differences in the excess cost of hospital service use for people living in the most deprived neighborhoods compared to those living in the least deprived neighborhoods: elective admissions (Female: 18% higher, Male: 16% higher), emergency admissions (Female: 62%, Male: 57%), maternity admissions (21%), outpatient appointments (Female: 18%, Male: 14%), and A&E attendances (Female: 68%, Male: 67%).

Another study from Kent, UK used individual patient level data aged 55 years and above to estimate the cost of inequality in primary and secondary care service use (16). The study found the inequality in health and social care costs of £109 million, equivalent to 15% of the estimated total expenditure in this age group. Our study estimated the age group specific cost of inequality in hospital service use and found that it contributed to 5.54% of the total expenses in people aged 65 years and above. However, different hospital service categories contributed differently to the cost of health inequality: A&E attendances contributed 26% of the total expenses, emergency admissions contributed 18.31%, outpatient appointments contributed 1.26% of the total expenses, but people from the least deprived neighborhoods uses higher elective admission services which results in negative percentage cost of (−4.58%) health inequality.

### Policy implications

The study findings have implications for resource allocation and development of strategies to reduce the gaps in health service utilization associated with socioeconomic inequality. Inequality in hospital service utilization for people living in more deprived neighborhoods compared to those living in the least deprived neighborhoods incur substantial avoidable costs to the NHS in Wales. We found that the higher cost of health inequality in emergency admissions and A&E attendances as a result of higher health service utilization from people living in more deprived neighborhoods which were to some extent counterbalanced by higher elective inpatient service use by older people from the least deprived neighborhoods.

Future work could look at how much care is used in appropriate parts of the system, for instance looking at the cost of admissions for ambulatory care sensitive conditions. There is evidence of greater needs in deprived neighborhoods, but this need is not necessarily materializing in the right place and time in the treatment pathway.

Evidence suggest that people from more deprived neighborhoods tend to visit health facilities more frequently due to poor health and comorbidity, which may create more pressure on the emergency service departments and primary care/GP services and thereby receive worse quality healthcare by these people. In addition, people living in the least deprived neighborhoods could be healthier and tend to manage planned visit at hospital and specialist care (14, 17). The *inverse care law* propounded by Tudor Hart in 1971 appropriately explained this situation (13).

Several efforts have been made at global, regional and national level to improve health and reduce health inequalities. Reducing health inequality is an integral part of WHO's new strategy (Health 2020) for better health in Europe through promotion of multi-sectoral and whole-of-government approaches. The regional office delivers wide range of activities and provides technical assistance to the member countries through various means to reduce such inequalities, including capacity building and learning exchanges, exploring evidence, promoting gender responsive policies, improving health of vulnerable groups, strengthening subnational and local governance (18). However, there is still a clear east-west gradient in health outcomes, with western countries performing relatively better compared to eastern counterpart in the WHO European Region (19). In Wales, There is also a wide equity gap in UK, including Wales, meaning that not much progress has been made since the “Inverse Care Law” was first coined in 1971 (4, 13). The equity gap in hospital service utilization resulting from existing healthcare arrangement could be addressed by more integrated healthcare, including better primary care (14). To address socio-economic inequality, the Welsh Government endorsed “A More Equal Wales” agenda, a statutory guidance based on Equality Act 2010 in March 2021, which aims to encourage public bodies for better decision making and to deliver better outcomes to the socio-economically disadvantaged groups (20).

Reducing the health equity gap is a complex multi-faceted multi-level challenge which requires tailored understanding and coherent action across different sectors, so we can (a) address the wider determinants of health inequalities; as well as (b) see what the healthcare service (NHS), social care and community services can do to tackle them (21, 22). A standardized case mix approach is an important consideration for the planning of healthcare resource use (23), as age, sex, and levels of deprivation have a significant impact on how and what services are used by the local community, which in turn impacts on the financial cost of delivering them (24). Community-based interventions targeted to those most in need could improve health outcomes (25), enable timely access to services (26), reduce hospital care need (27) and likely reduce inequalities (28) and thereby reducing healthcare costs, particularly associated with emergency and A&E contacts (27). Improving access/utilization of inpatient care by disadvantaged people which would have some cost could dramatically reduce costs of emergency admission in this population more than offsetting this additional investment in inpatient care. Above and beyond costs, this strategy would also unlock significant health benefits in this population. The COVID-19 pandemic has exacerbated existing health inequalities and created new vulnerabilities (29), which requires careful consideration to ensure inequalities in access to, delivery and quality of healthcare services are not amplified further, but reduced as part of the NHS and wider sustainable recovery (30). These findings will contribute to exploring further the relationship between health status, healthcare needs, health service access, provision and utilization, and the associated financial cost in order to help decision-makers to tailor services and prioritize investment toward reducing the health equity gap in Wales and beyond.

Our analyses estimated the cost of health inequality with area-based socioeconomic deprivation in Wales. We included whole population in the study, irrespective of their age, gender and socioeconomic status, so it may be reasonable to generalize the study results to other developed nations with similar type of health systems and population characteristics.

## Data availability statement

The original contributions presented in the study are included in the article/supplementary material, further inquiries can be directed to the corresponding author.

## Author contributions

RK has conceptualized and developed the manuscript and analyzed the data. JA and OD performed the quality check of the data and analysis work. RK, JA, OD, RM, BC, JC, MA, MD, MB, and RC subsequently revised the manuscript and approved the final draft. All authors contributed to the article and approved the submitted version.

## Funding

RC is supported by the Wellcome Trust (Grant No. 205427/Z/16/Z).

## Conflict of interest

The authors declare that the research was conducted in the absence of any commercial or financial relationships that could be construed as a potential conflict of interest.

## Publisher's note

All claims expressed in this article are solely those of the authors and do not necessarily represent those of their affiliated organizations, or those of the publisher, the editors and the reviewers. Any product that may be evaluated in this article, or claim that may be made by its manufacturer, is not guaranteed or endorsed by the publisher.
